# Trends of racial and ethnic disparities in virologic suppression among women in the HIV Outpatient Study, USA, 2010-2015

**DOI:** 10.1371/journal.pone.0189973

**Published:** 2018-01-02

**Authors:** Angelica Geter, Madeline Y. Sutton, Carl Armon, Marcus D. Durham, Frank J. Palella, Ellen Tedaldi, Rachel Hart, Kate Buchacz, on behalf of the HIV Outpatient Study Investigators

**Affiliations:** 1 Epidemiology Branch, Division of HIV/AIDS Prevention, Centers for Disease Control and Prevention, Atlanta, GA, United States of America; 2 Cerner Corporation, Kansas City, MO, United States of America; 3 Infectious Diseases Division, Northwestern University Feinberg School of Medicine, Chicago, IL, United States of America; 4 Department of Internal Medicine, Lewis Katz School of Medicine at Temple University, Philadelphia, PA, United States of America; University of Cincinnati College of Medicine, UNITED STATES

## Abstract

In the United States, women accounted for 19% of new HIV diagnoses in 2015 and were less likely to reach virologic suppression when compared to men. We assessed trends and disparities in virologic suppression among HIV-positive women to inform HIV treatment strategies. Data were from a prospective cohort of the HIV Outpatient Study and collected at nine United States HIV clinics. We included women aged ≥18 years, with ≥1 visit, who were prescribed antiretroviral therapy, and had ≥1 viral load test performed between 2010 and 2015. We defined virologic suppression as viral load <50 copies/mL and calculated adjusted prevalence ratios (aPR) with 95% confidence intervals (CI) for virologic suppression by race/ethnicity and year of measure. Generalized estimating equations were used for multivariable analyses to assess factors associated with virologic suppression. Among 809 women (median age = 44 years), 482 (60%) were black, 177 (22%) white, 150 (19%) Hispanic/Latina. Virologic suppression was less prevalent among black women (73%) compared with Hispanic/Latina women (83%) and white women (91%). In multivariable analyses, not achieving virologic suppression was more likely among black women (aPR = 2.13; CI = 1.50–3.02) or Hispanic/Latina women (aPR = 1.66; CI = 1.08–2.56) compared with white women, and among women who attended public clinics (aPR = 1.42; CI = 1.07–1.87) compared with those who attended a private clinic. Between 2010 and 2015, virologic suppression among HIV-positive women increased from 68% to 83%, but racial/ethnic disparities persisted. Black and Hispanic/Latina women had significantly lower rates of virologic suppression than white women. Interventions targeting virologic suppression improvement among HIV-positive women of color, especially those who attend public clinics, are warranted.

## Introduction

Since 1981, in the U.S. more than 170,000 women have been diagnosed with and more than 80,000 have died from HIV infection. Women comprised 24% of persons diagnosed with HIV in 2014 and 19% of persons diagnosed with HIV in 2015[1–3]. Although HIV diagnoses have declined among black/African American (hereafter referred to as black) and Hispanic/Latina women, these populations remain disproportionately affected by HIV, with black women accounting for 67% and Hispanic/Latina women accounting for 18% of all new HIV infections among women [2]. Improving the overall health of women and men diagnosed with HIV infection is a public health priority in the National HIV/AIDS Strategy (NHAS) [4–6]. Advances in antiretroviral therapy (ART) have improved the health of persons diagnosed with HIV, yet racial/ethnic disparities along the HIV care continuum (diagnosis, linkage/retention in care, ART prescriptions/medication adherence, and viral suppression) have persisted [4].

In 2013, among women diagnosed with HIV, an estimated 55% were retained in care, 39% were prescribed ART, and 30% achieved viral suppression; these estimates fell short of the NHAS target indicators for 90% of diagnosed persons to be retained in care and for 80% of diagnosed persons to be virally suppressed [3]. Black and Hispanic/Latina women were less likely to have access to care and HIV treatment services [3,4], and this likely contributes to higher morbidity and mortality by decreasing opportunities for some women to receive ART or reach an undetectable viral load [7–9]_._ Undetectable viral loads are essential to improving the overall health of persons diagnosed with HIV infection and reducing ongoing HIV transmission.

Because women of color are disproportionately affected by HIV infection, recent updates to NHAS (through 2020) included a focus on this population, to support and strengthen access to HIV treatment and care, increase viral suppression, and decrease HIV-related disparities [5]. Limited published reports have addressed disparities along the continuum of care, particularly for HIV-positive women of color [10]. To address this gap in the literature and examine indicators of progress along the continuum for women, we reviewed 2010 to 2015 trends in racial/ethnic disparities in viral suppression, among black, white, and Hispanic/Latina women using dynamic longitudinal cohort data from the HIV Outpatient Study (HOPS).

## Materials and methods

### The HIV Outpatient Study

The HOPS is an ongoing prospective observational cohort study of HIV-infected adults receiving care at nine HIV clinics (university-based, public and private) in six U.S. cities (Chicago, IL; Denver, CO; Long Island, NY; Philadelphia, PA; Tampa, FL; and Washington, DC) since 1993. The HOPS consists of an open cohort of patients who participate in the study post diagnosis of HIV infection and have the option to leave the study at any time [11]. Patient data, including socio-demographic characteristics, symptoms, diagnoses, treatments, and laboratory values are abstracted from medical charts and entered in an electronic database (Discover; Cerner Corporation, Kansas City, MO) by trained staff. These data are reviewed for quality and analyzed centrally. Through September 2016, the HOPS has collected information on more than 10,700 patients at over 508,000 clinical encounters. Since its inception, the HOPS protocol has been reviewed and approved annually by the institutional review board of the Centers for Disease Control and Prevention (CDC) (Atlanta, GA), Cerner Corporation (Kansas City, MO), and each local site’s institutional review board. The study protocol conforms to the guidelines of the U.S. Department of Health and Human Services for the protection of human subjects in research. The present analysis is based on the HOPS data current through September 30, 2016.

### Study population

**Fig 1:**
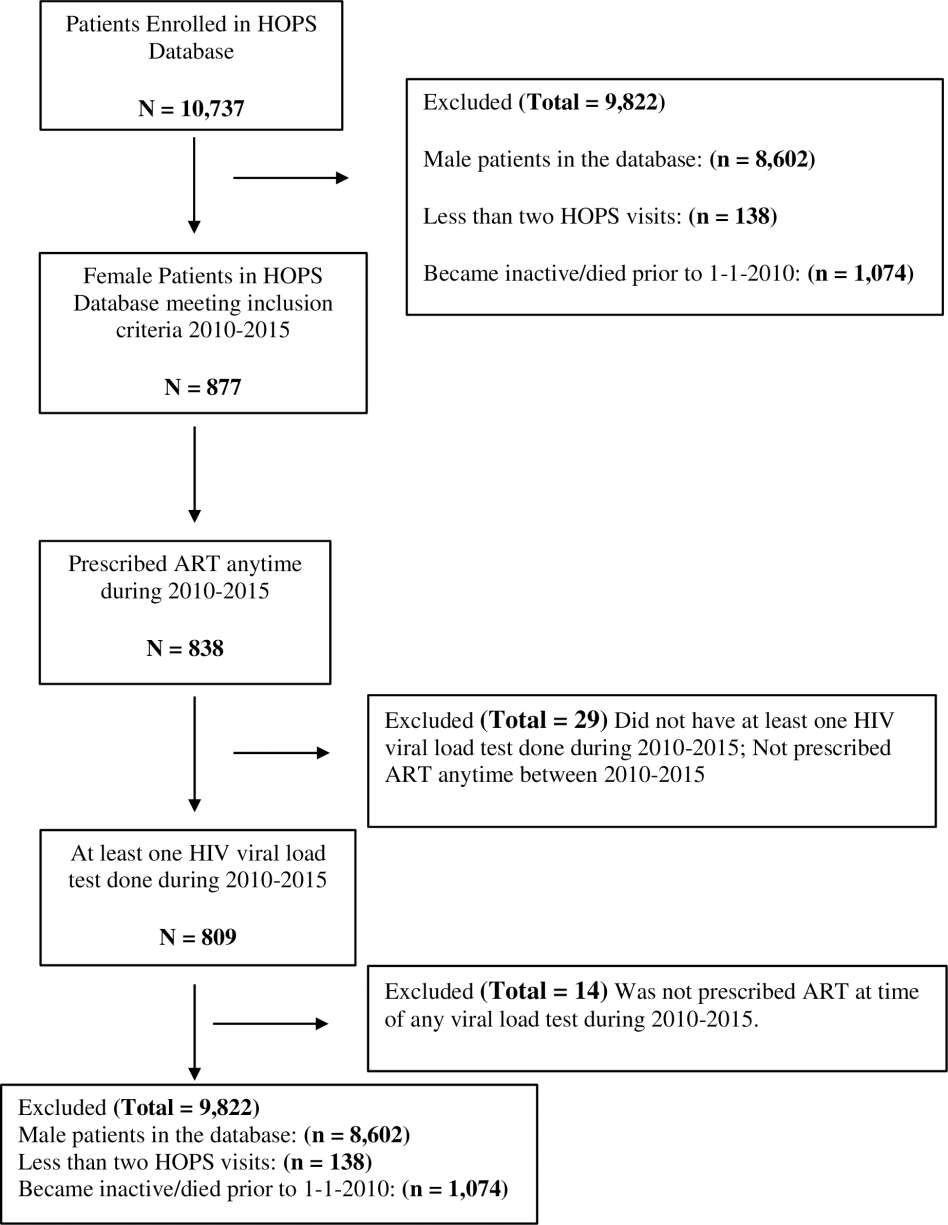
Selection steps flowchart of HOPS participants included in the analysis.

We analyzed data on 809 female HOPS participants with at least two HOPS visits since cohort inception in 1993, were seen anytime during 2010–2015, had either white, black, or Hispanic/Latina race/ethnicity, were prescribed ART anytime during 2010–2015, and had at least one viral load test done during this same time period (Fig 1).

### Measurements and definitions

The main outcome of interest was viral suppression, defined as having viral load < 50 copies/mL; an outcome based on the last viral load measurement of each calendar year of observation. The baseline (available only at HOPS entry) predictor variables included race/ethnicity (white, black, or Hispanic/Latina), education level (high school or less, any college, or unknown), employment status (full/part-time, or not employed or unknown), clinic type (public or private).

The following measures were assessed at study entry and at follow-up visits: age, insurance (private, public, or none), range in year of HIV diagnosis (< 2000, or 2000 or later), presence of an AIDS diagnosis (“AIDS” is in terms of a clinical [AIDS OI diagnosis] or immunologic [CD4 <200 cells/mm3] diagnosis) [12], viral load at baseline and follow-up (last clinical assessment or measurement during the observation time period), CD4+ cell count baseline and follow-up (closest to last viral load test in each calendar year when ARVs prescribed), and any ART exposure at viral load test date.

### Statistical analyses

Descriptive summaries of the data, and univariate and multivariable logistic and linear regression model analyses were done using SAS version 9.4 (SAS Institute, Cary, NC). Likelihood ratio chi-square or Fisher exact test were used to compare patient characteristics (binary or class variables) and Kruskal-Wallis or Wilcoxon rank-sum test were used to compare continuous variables by race/ethnicity. Proportion confidence intervals were obtained using the OpenEpi version 3.01 Mid-P Exact Method (Emory University, Rollins School of Public Health, Atlanta, GA). Univariate and multivariable analyses were performed using generalized estimated equation modeling assuming a binomial distribution. The effect measures were prevalence ratio for univariate analyses, and adjusted prevalence ratio for multivariable analyses. Results with p < 0.05 were considered statistically significant.

## Results

Among 809 women included in this analysis, 59% were black, 21% were white, 18% were Hispanic/Latina, the median age was 45 years, and 23% had private insurance. The median CD4+ cell count of the population was 463 cells/mm^3^, and 58% had a documented AIDS diagnosis. Eighty-four percent were prescribed ART (at the time of baseline viral load measurement), and the median duration of ART use was 7.1 years. In univariate analyses of baseline data, compared with white women, black and Hispanic/Latina women were significantly (p < 0.05) less ART-experienced but more likely to be publicly insured and cared for at public clinic (Table 1). Furthermore, black and Hispanic/Latina women had lower current CD4+ cell count, but similar nadir CD4+ cell count, compared with white women; black and Hispanic/Latina women were also less likely to be prescribed ART at the time of baseline viral load measurement or to have had any ART exposure at baseline (Table 1).

**Table 1:** Characteristics of women participants at baseline†, by race/ethnicity, the HIV Outpatient Study, 2010–2015 (N = 809).

Characteristics	White Women (n = 177)	Black Women (n = 482)	Hispanic/Latina Women (n = 150)	P-value*
**Age, years: median (IQR)**	45 (39–53)	44 (37–52)	44 (37–50)	0.12
**Education level: n (%)**				< 0.001
**High school or less**	67 (39.2)	265 (57.6)	95 (66.9)	
**Any college**	84 (49.1)	140 (30.4)	29 (20.4)	
**Unknown**	26 (14.7)	77 (16.0)	26 (17.3)	
**Employment status: n (%)**				0.007
**Full or part-time**	79 (44.6)	141 (29.3)	38 (25.3)	
** Not employed or unknown**	98 (55.4)	341 (70.7)	112 (74.7)	
**Insurance payer: n (%)**				< 0.001
**Private**	67 (37.9)	86 (17.8)	29 (19.3)	
**Public**	95 (53.7)	360 (74.7)	116 (77.3)	
**None/other/unknown payer**	15 (8.5)	36 (7.5)	5 (3.3)	
**Clinic type: n (%)**				< 0.001
**Public**	78 (44.1)	338 (70.1)	111 (74.0)	
**Private**	99 (55.9)	144 (29.9)	39 (26.0)	
**Years living with HIV: median (IQR)**	15.9 (9.6–19.4)	11.0 (3.7–16.7)	9.7 (3.8–14.9)	< 0.001
**IDU HIV risk**	31 (17.5)	45 (9.3)	11 (7.3)	0.006
**AIDS diagnosis: n (%)**	109 (61.6)	283 (58.7)	81 (54.0)	0.38
**CD4+ cell count/mm**^**3**^**: median (IQR) (n = 788)**	511 (327–804)	451 (261–720)	422 (276–711)	0.031
**Nadir CD4+ cell count/mm**^**3**^**: median (IQR) (n = 788)**	200 (50–364)	216 (80–350)	220 (83–333)	0.67
**Viral load <50 copies/mL: n (%)**	128 (72.3)	239 (49.6)	76 (50.7)	< 0.001
**ART use, years: median (IQR) (n = 676)**	10.8 (4.6–14.0)	5.9 (2.2–10.0)	7.5 (2.0–12.0)	< 0.001
**ART prescribed: n (%)**	165 (93.2)	391 (81.1)	120 (80.0)	< 0.001

†Table includes baseline data only (at HOPS study entry).

Abbreviations: ART, antiretroviral therapy; IDU, intravenous drug use; IQR, interquartile range.

* Likelihood ratio chi-square or Fisher exact test for binary or class variables, and Kruskal-Wallis or Wilcoxon rank-sum test for continuous variables

Patient characteristics for each average annual time point from 2010–2015 are summarized in Table 2. Of note, the number of women prescribed ART increased significantly between 2010 and 2015. Additionally, the percentage of women who were virally suppressed during the year increased significantly from 63% in 2010 to 77% in 2015 (Table 2). When stratified by age groups, data show a gradual increase in viral suppression for each age group over time (data not shown).

**Table 2:** Characteristics of women receiving HIV Care, by year, the HIV Outpatient Study, 2010–2015 (N = 809).

Characteristics	2010	2011	2012	2013	2014	2015
	n = 610	n = 637	n = 644	**n = 599**	**n = 576**	**n = 535**
**Age, years at last viral load during observation: median (IQR)**	46 (39–52)	46 (40–53)	47 (41–54)	48 (42–55)	49 (42–55)	49 (43–56)
Race/ethnicity: n (%)*						
**White**	153 (25.1)	152 (23.9)	148 (23.0)	135 (22.5)	129 (22.4)	111 (20.7)
**Black**	349 (57.2)	374 (58.7)	382 (59.3)	361 (60.3)	339 (58.9)	321 (60.0)
**Hispanic/Latina**	108 (17.7)	111 (17.4)	114 (17.7)	103 (17.2)	108 (18.8)	103 (19.3)
Education level: n (%)*						
**High school or less**	298 (48.9)	325 (51.0)	339 (52.6)	320 (53.4)	311 (54.0)	296 (55.3)
**Any college**	216 (35.4)	207 (32.5)	200 (31.1)	180 (30.1)	170 (29.5)	152 (28.4)
**Unknown**	96 (15.7)	105 (16.5)	105 (16.3)	99 (16.5)	95 (16.5)	87 (16.3)
Employment status: n (%)*						
**Full or part-time**	214 (35.1)	214 (33.6)	212 (32.9)	193 (32.2)	187 (32.5)	172 (32.1)
**Not employed or unknown**	396 (64.9)	423 (66.4)	432 (67.1)	406 (67.8)	389 (67.5)	363 (65.9)
Insurance payer: n (%)*						
**Private**	158 (25.9)	151 (23.7)	142 (22.0)	131 (21.9)	129 (22.4)	117 (21.9)
**Public**	413 (67.7)	444 (69.7)	460 (71.4)	429 (71.6)	412 (71.5)	393 (73.5)
**None/other/unknown payer**	39 (6.4)	42 (6.6)	42 (6.5)	39 (6.5)	35 (6.1)	25 (4.7)
Clinic type: n (%)*						
**Public**	373 (61.1)	413 (64.8)	432 (67.1)	420 (70.1)	413 (71.7)	398 (74.4)
**Private**	237 (38.9)	224 (35.2)	212 (32.9)	179 (29.9)	163 (28.3)	137 (25.6)
**Years living with HIV: median (IQR)**	12.6 (6.6–17.0)	12.1 (5.8–16.9)	11.3 (5.2–16.8)	11.0 (5.0–16.4)	10.5 (4.1–16.4)	9.8 (3.7–16.0)
IDU HIV risk*	74 (12.1)	77 (12.1)	70 (10.9)	64 (10.7)	65 (11.3)	57 (10.7)
**Clinical or immunologic AIDS: n (%)**†	394 (64.6)	396 (62.2)	402 (62.4)	374 (62.4)	368 (63.9)	339 (63.4)
**CD4+ cell count/mm3: median (IQR)**†	532 (322–785)	546 (334–800)	570 (350–852)	580 (376–853)	610 (382–913)	656 (423–954)
**Nadir CD4+ cell count/mm3: median (IQR)** †	194 (56–319)	213 (61–344)	208 (60–340)	211 (60–345)	201 (60–338)	203 (61–333)
**Viral load: median (IQR)**†	24 (24–146)	24 (10–104)	20 (10–69)	10 (10–38)	10 (10–35)	10 (10–10)
**Viral load <50 copies/mL: n (%)**†	416 (68.2)	449 (70.5)	456 (70.8)	458 (76.5)	464 (80.6)	444 (83.0)
**Viral load <50 copies/mL at all measurements in the year: n (%)**	381 (62.5)	416 (65.3)	439 (68.2)	417 (69.6)	436 (75.7)	414 (77.4)
**ART prescription, years (median IQR)**†	5.8 (0.6–10.6)	6.0 (0.8–11.1)	6.0 (0.9–11.7)	6.2 (0.8–12.3)	6.0 (1.0–12.9)	6.7 (1.6–13.8)
**ART prescribed: n (%)**†	583 (95.6)	611 (95.9)	626 (97.2)	587 (98.0)	571 (99.1)	534 (99.8)
**ART not prescribed: n (%)**†	27 (4.4)	26 (4.1)	18 (2.8)	12 (2.0)	5 (0.9)	1 (0.2)

Abbreviation: ART, antiretroviral therapy; IDU, intravenous drug use; IQR, interquartile range. Likelihood ratio chi-square or Fisher exact test for binary or class variables, and Kruskal-Wallis or Wilcoxon rank-sum test for continuous variables.

*Indicates baseline measurement (at HOPS study entry)

†Indicates measure at last clinical measurement for each time point

The 795 women who had at least one viral load measured and were prescribed ART contributed a total of 7,406 viral loads measured in the longitudinal (2010–2015) analyses. In the univariate generalized estimated equation models, factors associated with not achieving viral suppression included younger age, black or Hispanic/Latina race/ethnicity, having an education level of high school or less, having public insurance, receiving care in a public clinic, and having baseline CD4+ cell count < 350 cells/mm3 (all p < 0.05). In multivariable analyses, factors associated with not achieving viral suppression included: younger age, black or Hispanic/Latina race/ethnicity, receiving care in a public clinic, and having baseline CD4+ cell count < 350 cells/mm^3^ (all p < 0.05) (Table 3).

**Table 3:** Generalized estimating equation analyses of factors associated with not having viral suppression when prescribed ART, the HIV Outpatient Study, 2010–2015 (N = 795).

Patient Characteristics	Univariate model		Multivariable Full model		Multivariable Parsimonious	
	PR (95% CI)	P-value	aPR (95% CI)	P-value	aPR (95% CI)	P-value
Age, years*†				** **	** **	** **
**≤29**	2.13 (1.41–3.21)	< 0.001	2.81 (1.86–4.25)	< 0.001	2.77 (1.85–4.17)	< 0.001
**30–39**	1.56 (1.11–2.20)	0.011	2.15 (1.52–3.05)	< 0.001	2.16 (1.53–3.07)	< 0.001
**40–49**	1.38 (0.98–1.93)	0.06	1.68 (1.17–2.41)	0.004	1.66 (1.16–2.38)	0.006
**≥50**	Reference		Reference		Reference	
**Race/Ethnicity**†						
**White, non-Hispanic/Latina**	Reference		Reference		Reference	
**Black, non-Hispanic/Latina**	2.46 (1.74–3.48)	< 0.001	1.99 (1.39–2.85)	< 0.001	2.13 (1.50–3.02)	< 0.001
**Hispanic/Latina**	0.82 (0.58–1.15)	0.25	1.52 (0.98–2.36)	0.033	1.66 (1.08–2.56)	0.020
**Education level**						
**High school or less**	1.60 (1.20–2.14)	0.002	1.15 (0.85–1.55)	0.39		
**Any college**	Reference		Reference			
**Unknown**	1.16 (0.74–1.82)	0.52	0.80 (0.53–1.23)	0.28		
**Employment Status**						
**Full or part time**	Reference		Reference			
**Not employed or unknown**	1.29 (0.98–1.69)	0.07	1.13 (0.85–1.51)	0.42		
Insurance Payer*						
**Private**	Reference		Reference			
**Public**	1.45 (1.06–1.99)	0.021	1.09 (0.77–1.53)	0.64		
**None/Other/Unknown payer**	1.29 (0.98–1.69)	0.30	1.15 (0.69–1.90)	0.61		
**Clinic Type**†						
**Public**	1.60 (1.21–2.12)	< 0.001	1.34 (1.00–1.79)	0.10	0.70 (0.45–1.09)	0.11
**Private**	Reference		Reference		Reference	
**IDU HIV risk**†						
**Yes**	0.61 (0.40–0.93)	0.022	0.70 (0.45–1.07)	0.06	1.42 (1.07–1.87)	0.014
**No**	Reference		Reference		Reference	
CD4+ cell count < 350 cells/mm3*†						
**Yes**	3.96 (3.26–4.81)	< 0.001	4.32 (3.52–5.32)	< 0.001	4.31 (3.51–5.29)	< 0.001
**No**	Reference		Reference		Reference	** **

Abbreviations: aPR, adjusted prevalence ratio; ART, antiretroviral therapy; IDU, intravenous drug use; PR, prevalence ratio.

*Variables updated during follow-up (measured closest to last viral load test in each calendar year when ARVs prescribed).

† Variables selected for the parsimonious model had P-values ≤ 0.10 in the Multivariable Full Model.

**Fig 2:**
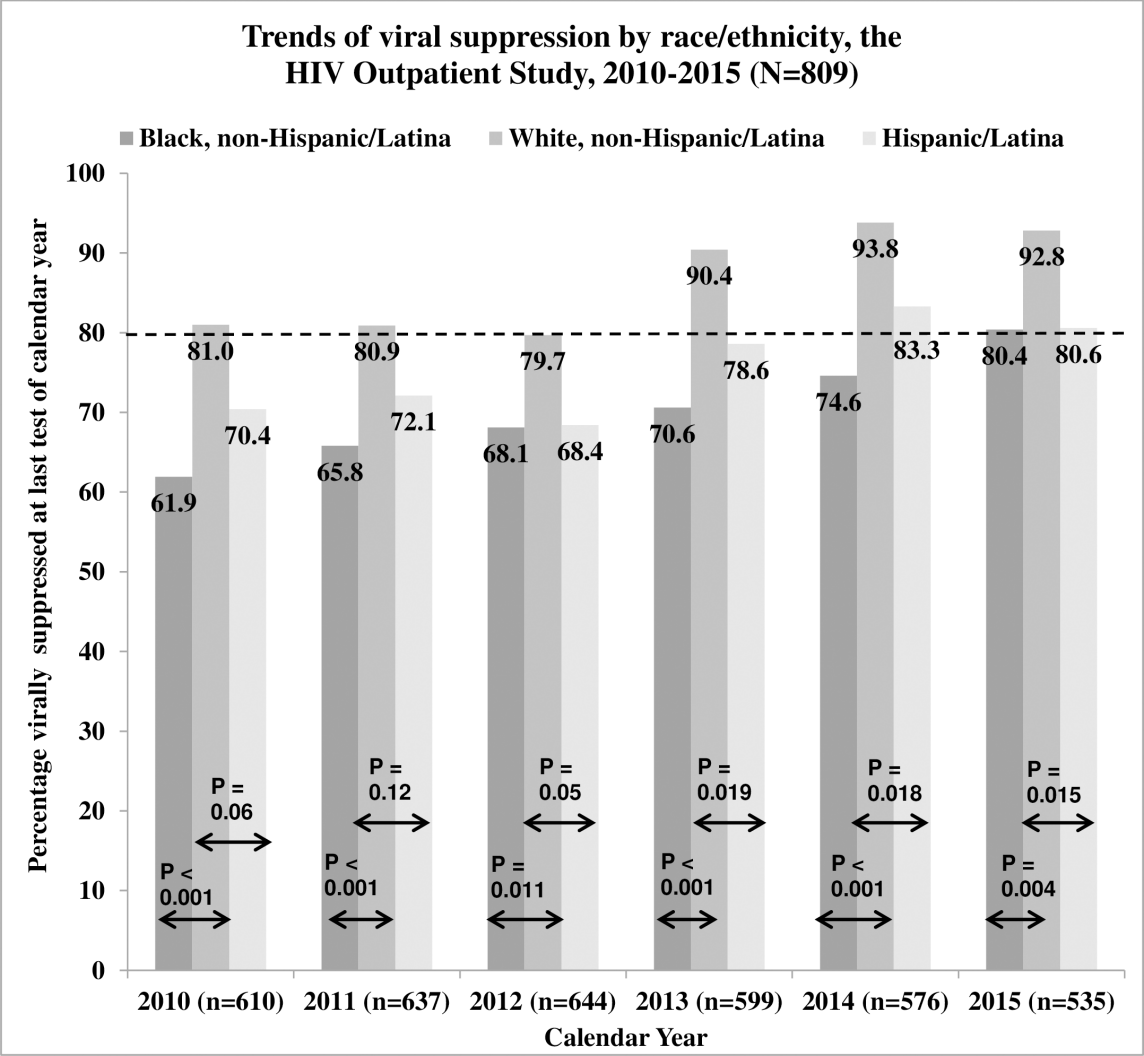
Trends of viral suppression among women by race/ethnicity, the HIV Outpatient Study, USA, 2010–2015 (N = 809). Proportion confidence intervals were obtained using the OpenEpi version 3.01 Mid-P Exact Method.

Significant black-white and Hispanic/Latina-white disparities in viral suppression existed for each year from 2010 through 2015 (p < 0.05). Rates of viral suppression for black and Hispanic/Latina women lag significantly behind the viral suppression rates for white women; however, by 2015, all racial/ethnic subgroups of women engaged in HOPS care met or exceeded the NHAS viral suppression indicator of 80% among HIV-diagnosed persons (Fig 2).

**Fig 3:**
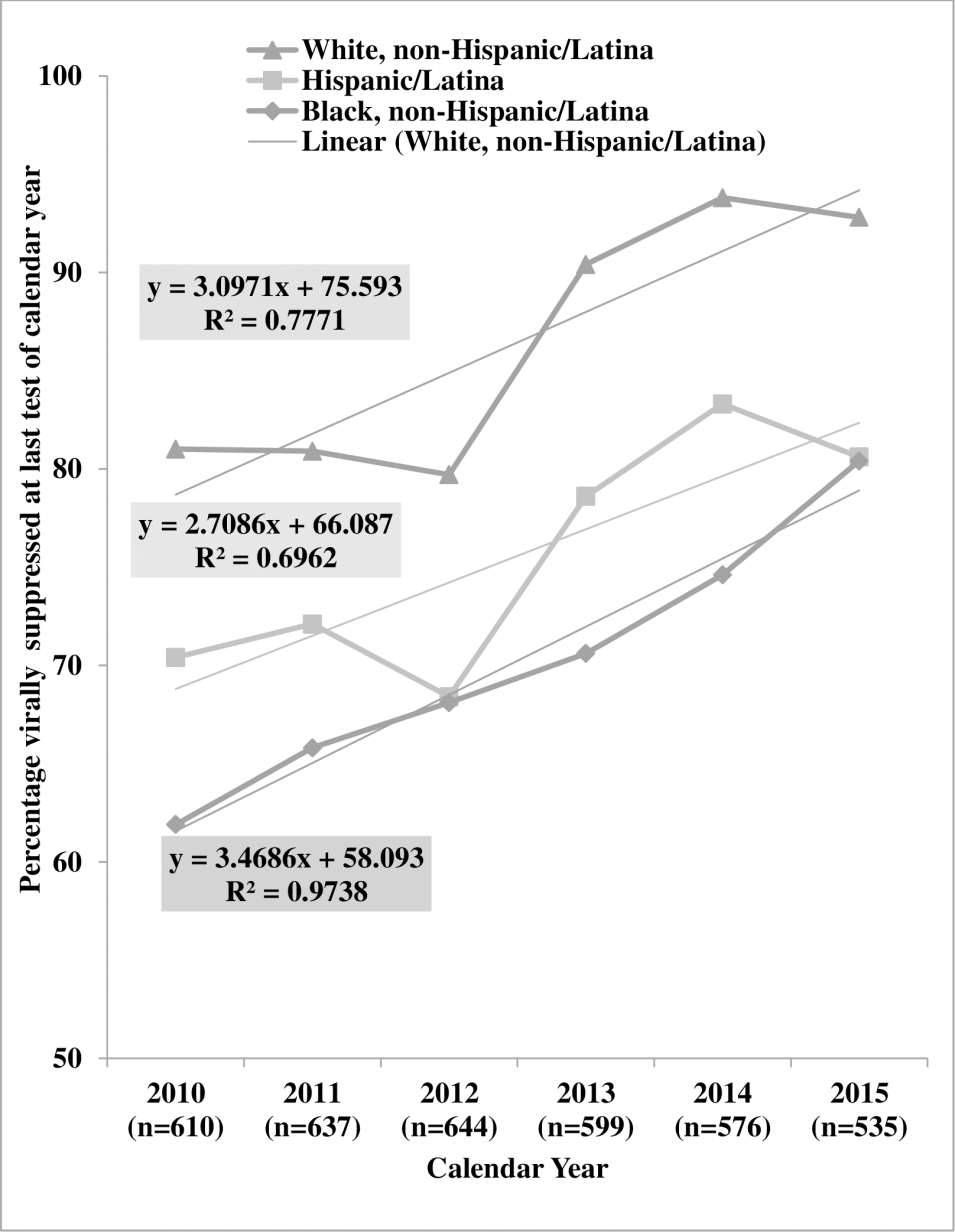
Trends of viral suppression by race/ethnicity, the HIV Outpatient Study, 2010–2015 (N = 809).

Pairwise comparisons of regression line slopes indicated no significant differences by race/ethnicity: white women vs Hispanic/Latina women (p = 0.76), white women vs black women (p = 0.68), and Hispanic/Latina women vs black women (p = 0.44). Pairwise comparisons of regression line intercepts indicated a significant difference white and black women: white women vs Hispanic/Latina women (p = 0.08), white women vs black women (p < 0.001), Hispanic/Latina vs black women (p = 0.06). (Fig 3)

## Discussion

We found that among all women in the HOPS, ART use increased from 2010 to 2015, and there was a corresponding and upward trend in viral suppression during this time. However, despite improvements in viral suppression among all women year to year, racial/ethnic disparities persisted from 2010–2015. These findings are consistent with previous cross-sectional reports showing gender-based VS disparities, and racial/ethnic disparities in VS among women, with increased VS suppression trends mostly mediated by increases in ART prescriptions [10]. Earlier analyses by Beer et al. also reported annual increases in viral suppression among male and female persons living with HIV in care, with decreasing–yet persistent–racial/ethnic disparities noted among black MSM [13]. Based on our review of the literature, our study is one of the first published reports of a trends analysis of viral suppression with a diverse group of women living with HIV infection who are part of a dynamic longitudinal cohort. Thus, our analysis contributes to the sparse body of knowledge regarding trends in racial/ethnic disparities in viral suppression and supports the need for ongoing research among HIV-positive women to strengthen HIV prevention strategies for women.

We found that not having viral suppression after controlling for ART prescription was associated with younger age (< 50 years), black or Hispanic/Latina race/ethnicity, being seen in a public clinic, and having baseline CD4+ cell count < 350 cells/mm^3^. These factors are closely aligned with recent reports suggesting that women were more likely to be virally suppressed if they had uninterrupted access to ART through either private insurance [14], the AIDS Drug Assistance Program [14–15], or Ryan White HIV/AIDS Programs [16]. These data all suggest that increased clinical efforts to maintain adherence and reach viral suppression with women may be warranted, especially with younger women or those who initially present for care with lower CD4 cell counts.

Prior HOPS cohort analyses have found poorer health outcomes among publically vs. privately insured patients [17] and lower CD4 cell counts soon after HIV diagnosis among HOPS patients accessing care at public vs. private clinics [18], findings that appeared related to systematic differences in the characteristics of patients accessing care at private vs. public clinics. However, lower viral suppression rates among HIV-positive women who attended public clinics in our study also suggests that a closer examination of clinical and social supports at public clinics may be warranted. Many public clinics have resource challenges due to decreased staff, high clinical volume and long waits that may be discouraging for some patients, especially for some women who often have competing priorities and more constraints on their time due to work and family [19]. Some published studies suggest that flexible clinical time schedules, non-judgmental and sensitive staff persons, comprehensive access at a single location, and options for community-based access all provide options that facilitate HIV care for women living with HIV infection especially Black and Hispanic women/Latinas [20, 21]. Notably, recent national analyses have found that HIV patients served in clinics receiving Ryan White-supported care can experience improvements in health outcomes that parallel or exceed those seen for patients served in other settings [22].

We found significant black-white and Hispanic/Latina-white disparities in viral suppression for each year from 2010 through 2015, although each racial/ethnic group reached the NHAS indicator of 80% viral suppression by 2015. This is encouraging; however, the lower rates of viral suppression among black and Hispanic/Latina women compared with white women suggest substantial barriers to HIV treatment and care still exist. Social and structural barriers to HIV treatment and care among black women have been noted. These barriers include access to and quality of HIV treatment services [23–26], lack of integrated care that includes spiritual, mental, and emotional wellness [26–29] and HIV-related stigma, particularly from healthcare providers [30–32]. Recommendations from a 2012 meeting of the President’s Advisory Council suggested that policy and practice changes were needed to reduce the domestic HIV epidemic on women, particularly women of color, developing interventions that removed structural barriers for women, and the inclusion of women living with HIV in research and implementation agendas, were discussed as top priorities [32]. Our findings support the ongoing need to identify, prioritize and address patient-level, social and structural barriers to accessing and adhering to HIV treatment and care among women of color.

Our study has some limitations. First, we analyzed data from nine HIV clinic sites (private practice and public/university-based practice) using a convenience sample of their HIV-infected patients in care. Therefore, our data may not be generalizable to women in areas not represented amongst our six clinical study locations. Second, data collection was limited to medical records of routine HIV care visits, and HOPS did not collect systematic information on psychosocial, economic and other social and structural determinants of health that could potentially explain the differences in viral suppression observed by race/ethnicity. Future studies can strengthen our findings by obtaining social determinants of health (SDH) information to enhance data interpretation and future intervention development. Data regarding barriers to HIV treatment and care among women of color are limited; surveillance systems, surveys, and interventions can be strengthened by collecting these SDH data from persons living with HIV infection and prioritizing women of color within their recruitment efforts [33–34]. These data should examine topics such as facilitators and barriers to prioritizing HIV treatment (e.g. social support, access to quality childcare, transportation, partner HIV status, and disclosure of HIV status), access and linkage to care, retention in care, economic stability, survival sex (i.e. exchanging sex for food and money), provider-patient communication, medical mistrust, and HIV-related stigma by healthcare providers and support systems. Third, it is important to note the possibility of misclassification of ARV experience, accounting for the possibility that patients may have received care in clinics not part of the HOPS sites. Fourth, we used a viral suppression definition based on the last viral load measurement of each calendar year of observation, which is subject to variability and imprecision. However, this measure has been used previously and validated in other studies that examined viral suppression [35]. Future studies can consider “sustained” viral suppression, which allows a measure of viral loads during the previous 12 months. Finally, although the HOPS is a convenience cohort of patients seen at nine U.S. HIV clinics, our population is demographically diverse and likely similar clinically to HIV-infected persons in care across the nation [36].

Although progress has been made along the HIV care continuum among black and Hispanic/Latina women, disparities in HIV treatment still exist. Interventions targeting viral suppression improvement among black and Hispanic/Latina women, and women who are younger or who attend public clinics, are warranted. Our findings underscore the importance of qualitative and quantitative research to develop HIV interventions that are gender-based, culturally tailored and address social and structural barriers in an effort to further close the viral suppression gaps among women living with HIV infection.
