# Correction: Trends of racial and ethnic disparities in virologic suppression among women in the HIV Outpatient Study, USA, 2010-2015

**DOI:** 10.1371/journal.pone.0194413

**Published:** 2018-03-12

**Authors:** Angelica Geter, Madeline Y. Sutton, Carl Armon, Marcus D. Durham, Frank J. Palella, Ellen Tedaldi, Rachel Hart, Kate Buchacz, on behalf of the HIV Outpatient Study Investigators

There is an error in the third sentence of the second paragraph in the Results section. The correct sentence is: Additionally, the percentage of women who were virally suppressed during the year increased significantly from 68% in 2010 to 83% in 2015 (Table 2).

There is an error in the second paragraph of the “Measurements and definitions” subsection of the Materials and methods section. The correct sentence is: The following measures were assessed at baseline for this cohort (2010–2015) and at follow-up visits: age, insurance (private, public, or none), range in year of HIV diagnosis (< 2000, or 2000 or later), presence of an AIDS diagnosis (“AIDS” is in terms of a clinical [AIDS OI diagnosis] or immunologic [CD4<200 cells/mm3] diagnosis) [12], viral load at baseline and follow-up (last clinical assessment or measurement during the observation time period), CD4+ cell count baseline and follow-up (closest to last viral load test in each calendar year when ARVs prescribed), and any ART exposure at viral load test date.

There is an error in the Author Contributions section. Carl Armon should be listed as the only author who contributed to formal analysis.

There are errors in Fig 1 and Fig 3. Please view the correct Fig 1 and Fig 3 here.

**Fig 1:**
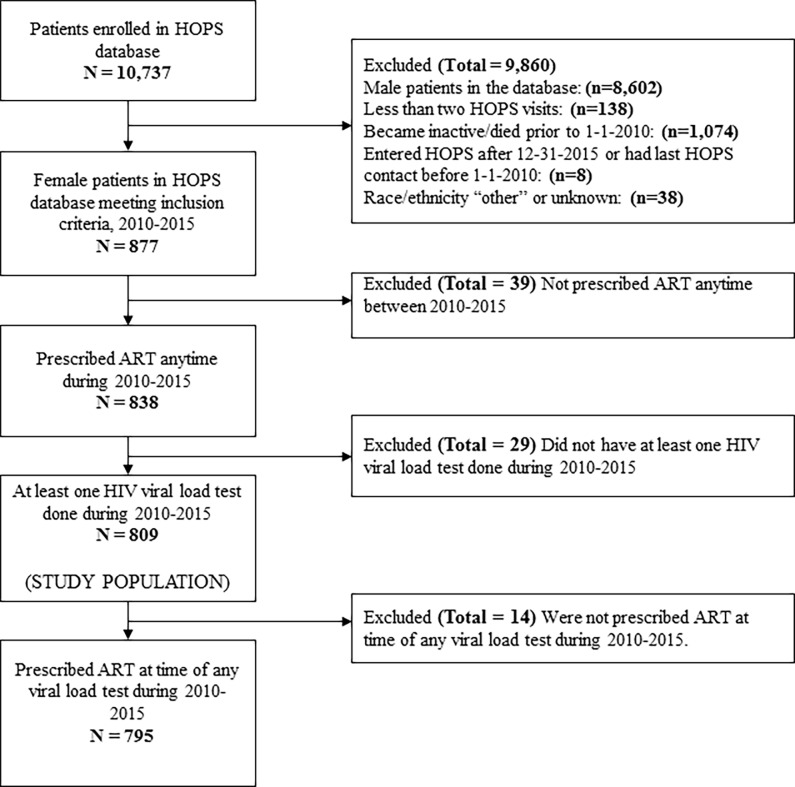
Selection steps flowchart of HOPS participants included in the analysis.

**Fig 3:**
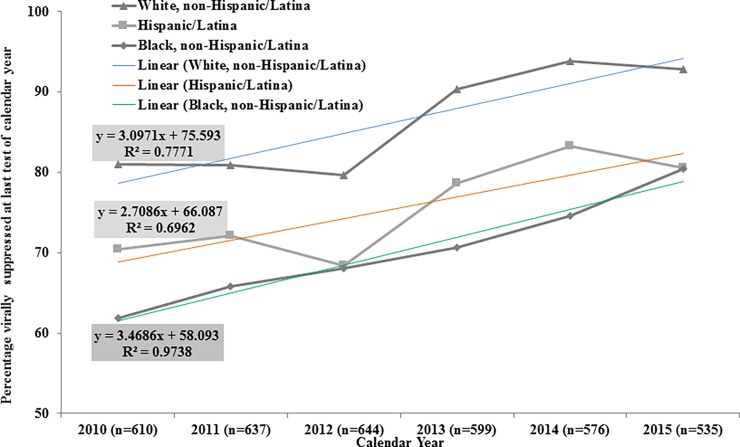
Trends of viral suppression by race/ethnicity, the HIV Outpatient Study, 2010–2015 (N = 809).

There are errors in Table 1. Please view the correct Table 1 here.

**Table 1:** Characteristics of Women Participants At Baseline†, by Race/Ethnicity, The HIV Outpatient Study, 2010–2015 (N = 809).

Characteristics	White Women (n = 177)	Black Women (n = 482)	Hispanic/Latina Women (n = 150)	P-value*
**Age, years: median (IQR)**	45 (39–53)	44 (37–52)	44 (37–50)	0.12
**Education level: n (%)**†				< 0.001
**High school or less**	67 (39.2)	265 (57.6)	95 (66.9)	
**Any college**	84 (49.1)	140 (30.4)	29 (20.4)	
**Unknown**	26 (14.7)	77 (16.0)	26 (17.3)	
**Employment status: n (%)**†				0.007
**Full or part-time**	79 (44.6)	141 (29.3)	38 (25.3)	
**Not employed or unknown**	98 (55.4)	341 (70.7)	112 (74.7)	
**Insurance payer: n (%)**				< 0.001
**Private**	67 (37.9)	86 (17.8)	29 (19.3)	
**Public**	95 (53.7)	360 (74.7)	116 (77.3)	
**None/other/unknown payer**	15 (8.5)	36 (7.5)	5 (3.3)	
**Clinic type: n (%)**†				< 0.001
**Public**	78 (44.1)	338 (70.1)	111 (74.0)	
**Private**	99 (55.9)	144 (29.9)	39 (26.0)	
**Years living with HIV: median (IQR)**	15.9 (9.6–19.4)	11.0 (3.7–16.7)	9.7 (3.8–14.9)	< 0.001
**IDU HIV risk**	31 (17.5)	45 (9.3)	11 (7.3)	0.006
**AIDS diagnosis: n (%)**	109 (61.6)	283 (58.7)	81 (54.0)	0.38
**CD4+ cell count/mm**^**3**^**: median (IQR) (n = 788)**	511 (327–804)	451 (261–720)	422 (276–711)	0.031
**Nadir CD4+ cell count/mm**^**3**^**: median (IQR) (n = 788)**	200 (50–364)	216 (80–350)	220 (83–333)	0.67
**Viral load <50 copies/mL: n (%)**	128 (72.3)	239 (49.6)	76 (50.7)	< 0.001
**ART use, years: median (IQR) (n = 676)**	10.8 (4.6–14.0)	5.9 (2.2–10.0)	7.5 (2.0–12.0)	< 0.001
**ART prescribed: n (%)**	165 (93.2)	391 (81.1)	120 (80.0)	< 0.001

Abbreviations: ART, antiretroviral therapy; IQR, interquartile range.

†Collected at HOPS study entry.

* Likelihood ratio chi-square or Fisher exact test for binary or class variables, and Kruskal-Wallis or Wilcoxon rank-sum test for continuous variables

There are errors in Table 3. Please view the correct Table 3 here.

**Table 3:** Generalized Estimating Equation Analyses of Factors Associated With Not Having Viral Suppression When Prescribed ART, The HIV Outpatient Study, 2010–2015 (N = 795).

Patient Characteristics	Univariate model		Multivariable Full model		Multivariable Parsimonious	
	PR (95% CI)	P-value	aPR (95% CI)	P-value	aPR (95% CI)	P-value
Age, years*^†^				** **	** **	** **
**≤29**	2.13 (1.41–3.21)	< 0.001	2.81 (1.86–4.25)	< 0.001	2.77 (1.85–4.17)	< 0.001
**30–39**	1.56 (1.11–2.20)	0.011	2.15 (1.52–3.05)	< 0.001	2.16 (1.53–3.07)	< 0.001
**40–49**	1.38 (0.98–1.93)	0.06	1.68 (1.17–2.41)	0.004	1.66 (1.16–2.38)	0.006
**≥50**	Reference		Reference		Reference	
**Race/Ethnicity**^**†**^						
**White, non-Hispanic/Latina**	Reference		Reference		Reference	
**Black, non-Hispanic/Latina**	2.46 (1.74–3.48)	< 0.001	1.99 (1.39–2.85)	< 0.001	2.13 (1.50–3.02)	< 0.001
**Hispanic/Latina**	0.82 (0.58–1.15)	0.25	1.52 (0.98–2.36)	0.033	1.66 (1.08–2.56)	0.020
**Education level**						
**High school or less**	1.60 (1.20–2.14)	0.002	1.15 (0.85–1.55)	0.39		
**Any college**	Reference		Reference			
**Unknown**	1.16 (0.74–1.82)	0.52	0.80 (0.53–1.23)	0.28		
**Employment Status**						
**Full or part time**	Reference		Reference			
**Not employed or unknown**	1.29 (0.98–1.69)	0.07	1.13 (0.85–1.51)	0.42		
Insurance Payer*						
**Private**	Reference		Reference			
**Public**	1.45 (1.06–1.99)	0.021	1.09 (0.77–1.53)	0.64		
**None/Other/Unknown payer**	1.29 (0.98–1.69)	0.30	1.15 (0.69–1.90)	0.61		
**Clinic Type**^**†**^						
**Public**	1.60 (1.21–2.12)	< 0.001	1.34 (1.00–1.79)	0.10	1.42 (1.07–1.87)	0.014
**Private**	Reference		Reference		Reference	
**IDU HIV risk**^**†**^						
**Yes**	0.61 (0.40–0.93)	0.022	0.70 (0.45–1.07)	0.06	0.70 (0.45–1.09)	0.11
**No**	Reference		Reference		Reference	
CD4+ cell count < 350 cells/mm3*						
**Yes**	3.96 (3.26–4.81)	< 0.001	4.32 (3.52–5.32)	< 0.001	4.31 (3.51–5.29)	< 0.001
**No**	Reference		Reference		Reference	** **

Abbreviations: aPR, adjusted prevalence ratio; ART, antiretroviral therapy; IDU, intravenous drug use; PR, prevalence ratio.

*Variables updated during follow-up (measured closest to last viral load test in each calendar year when ARVs prescribed).

^**†**^ Variables selected for the parsimonious model had P-values ≤ 0.10 in the Multivariable Full Model.
